# Supplementary material for: Cas12a2 elicits abortive infection through RNA-triggered destruction of dsDNA
Source: Nature. 2023 Jan 4;613(7944):588–94. doi: 10.1038/s41586-022-05559-3 (PMC9811890; doi:10.1038/s41586-022-05559-3)

nature portfolio

https://doi.org/10.1038/s41586-022-05559-3

Supplementary information

Cas12a2 elicits abortive infection through RNA-triggered destruction of dsDNA

In the format provided by the authors and unedited

a

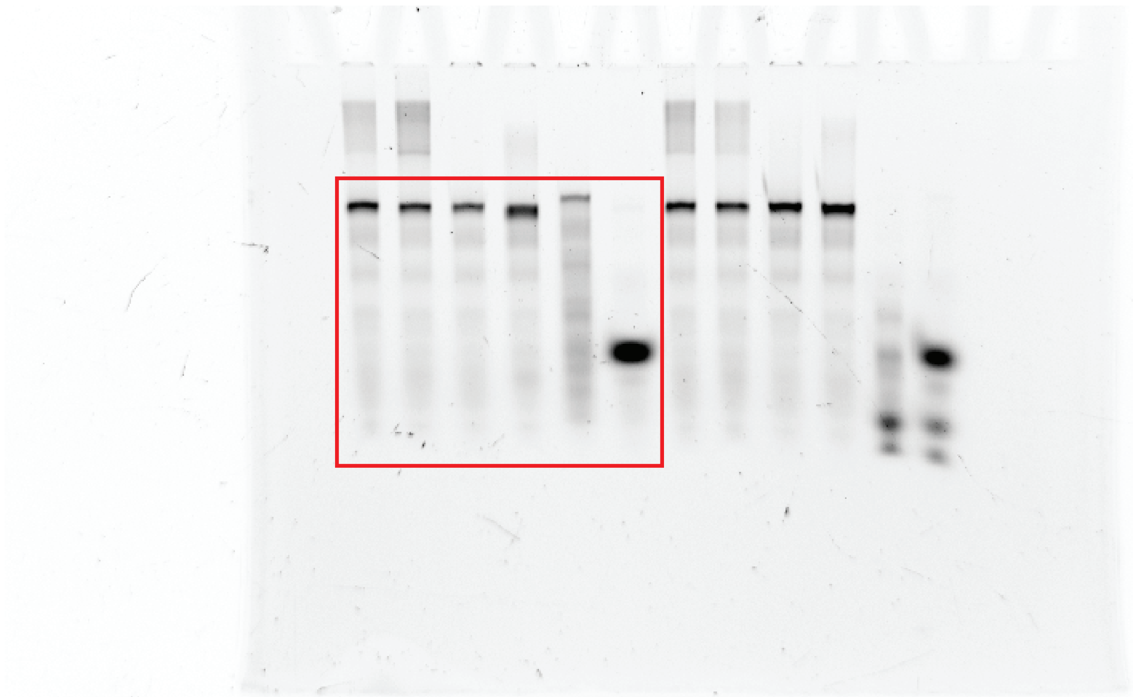

b

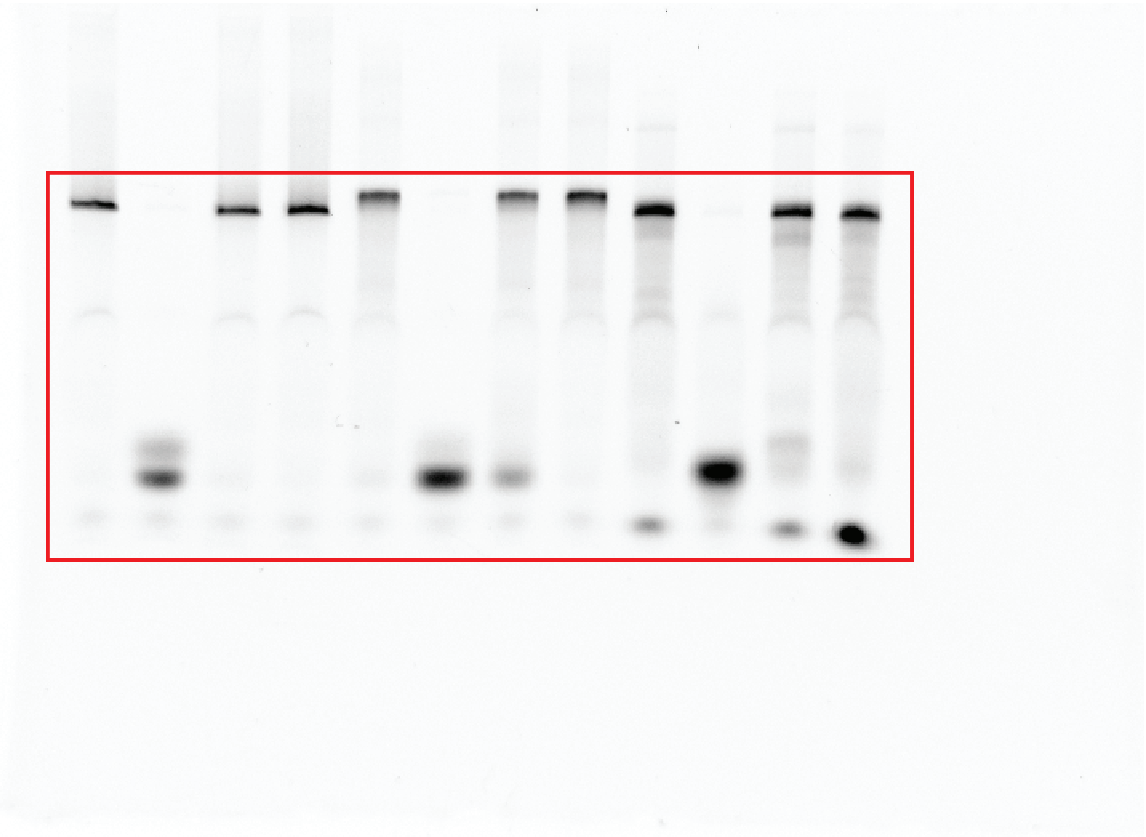

c

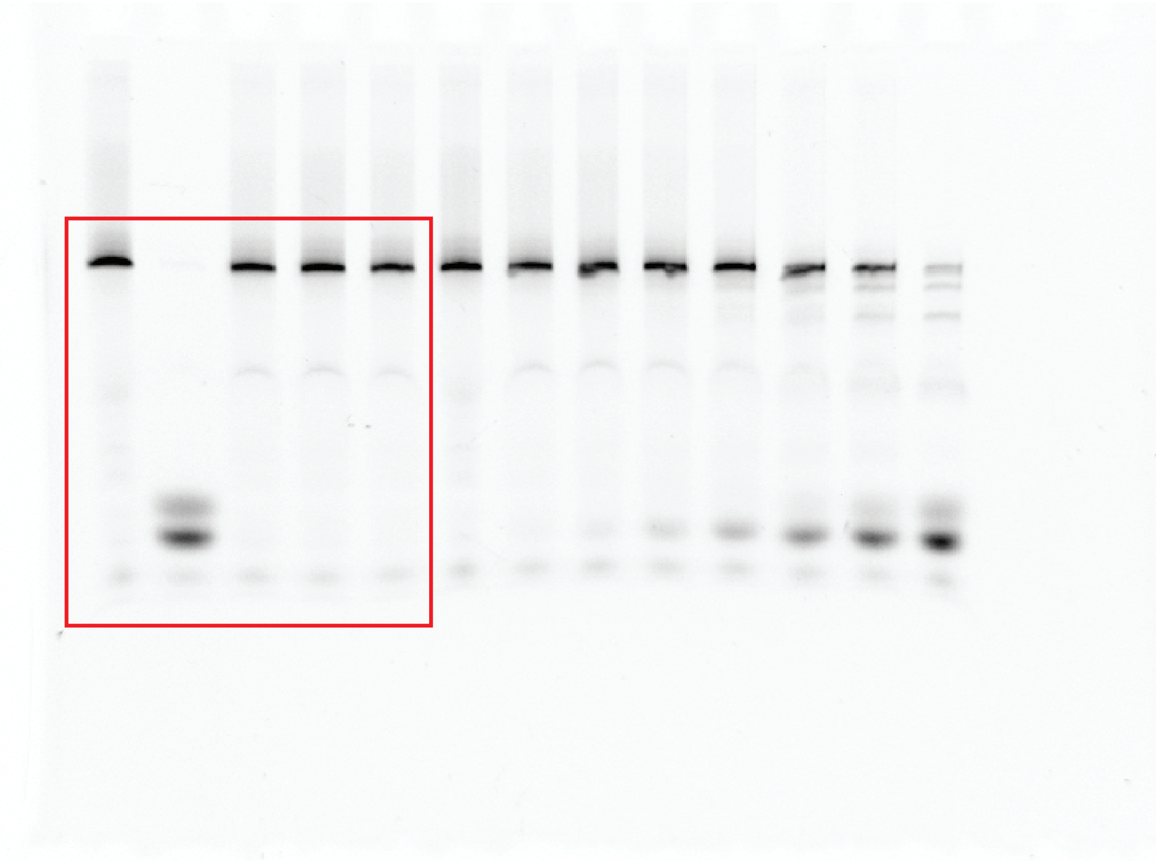

d

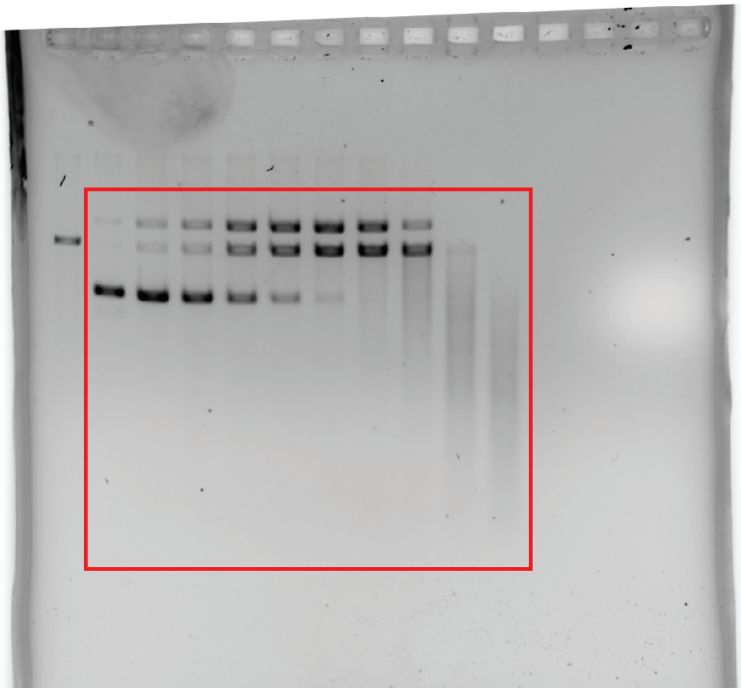

e

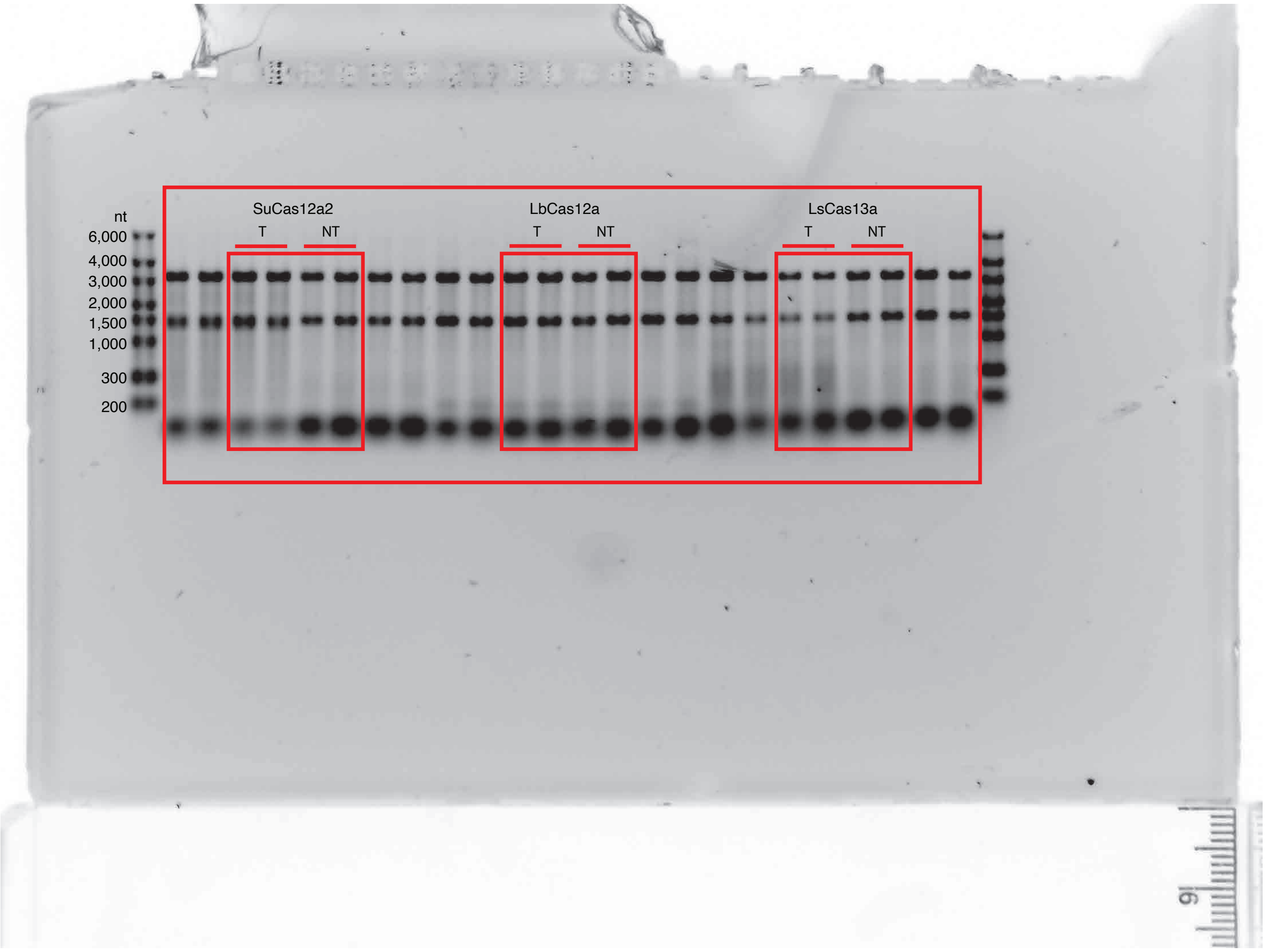
nt
6,000
4,000
3,000
2,000
1,500
1,000
300
200
SuCas12a2
T
NT
LbCas12a
T
NT
LsCas13a
T
NT

f

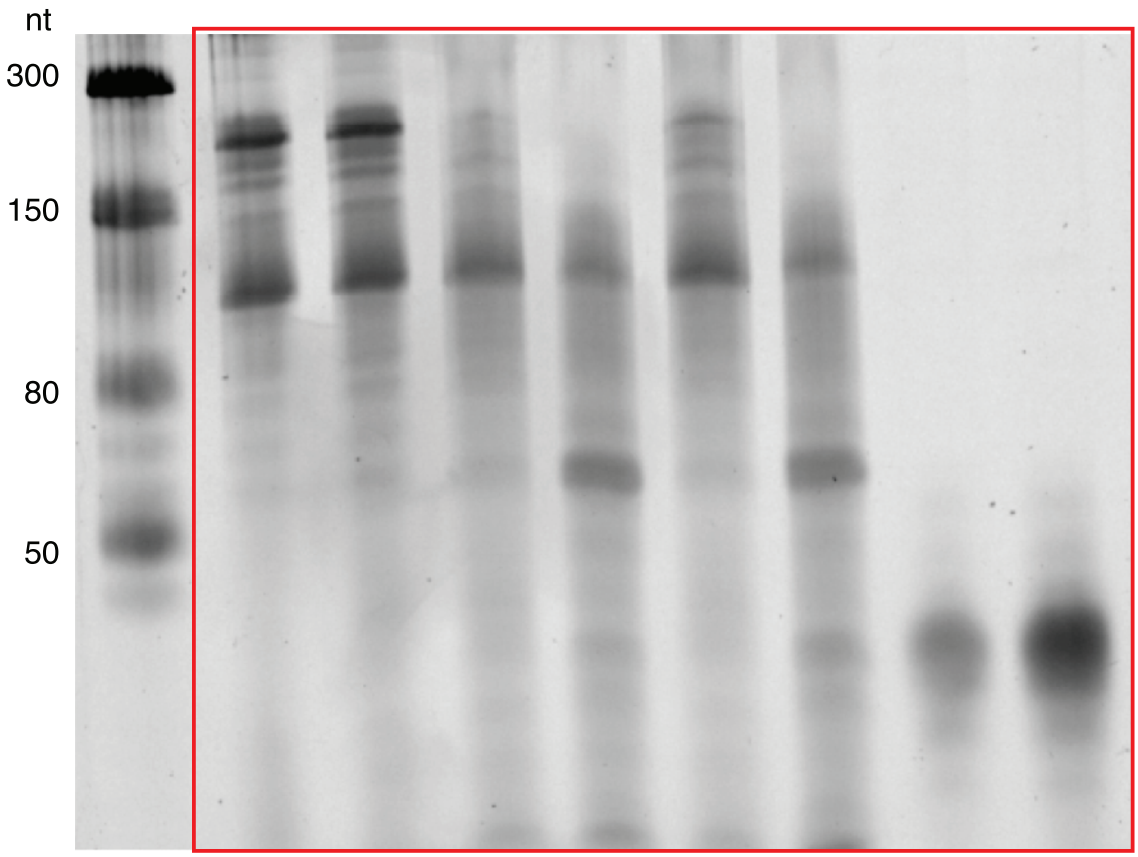

g

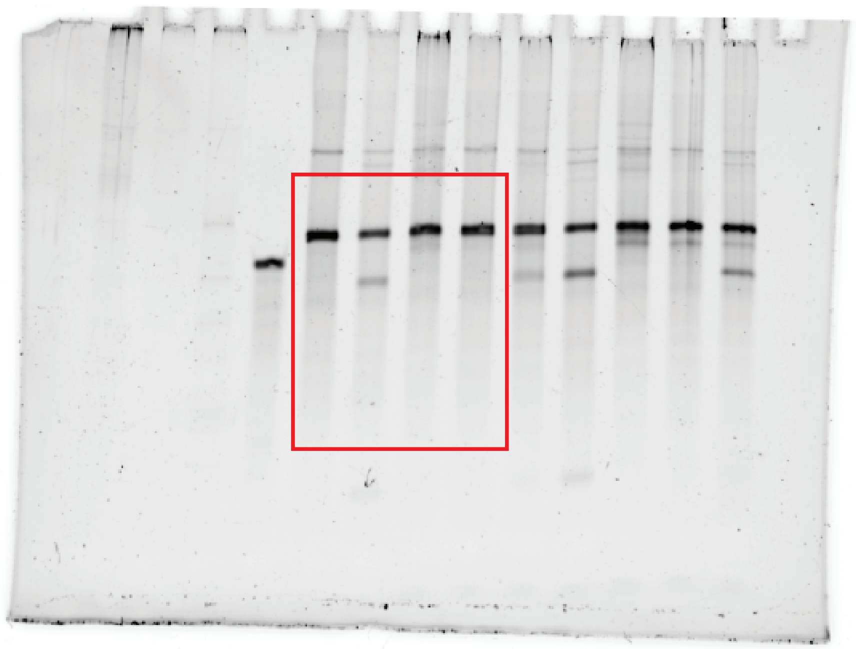

h

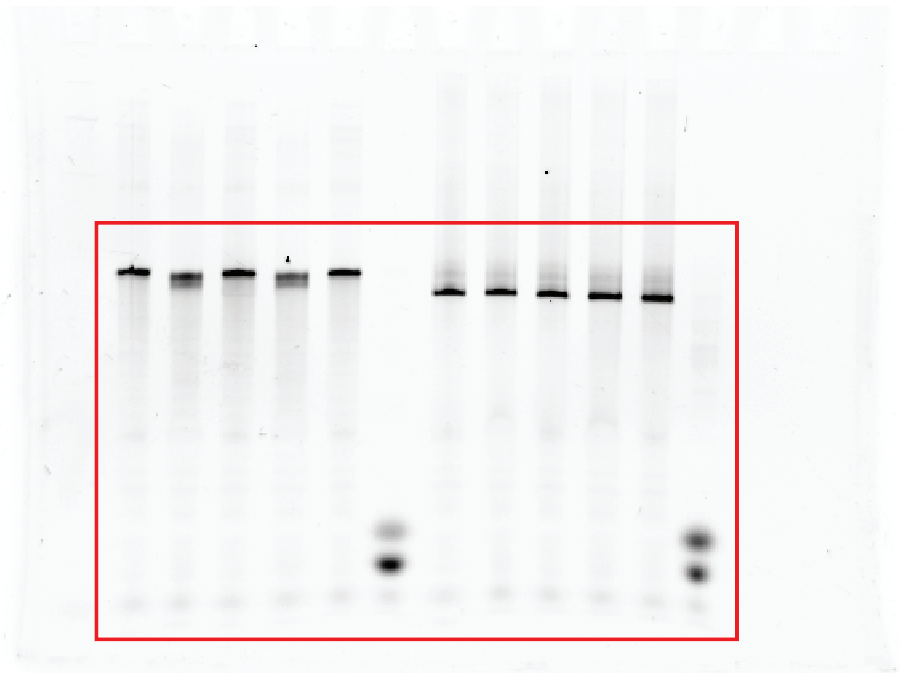

i

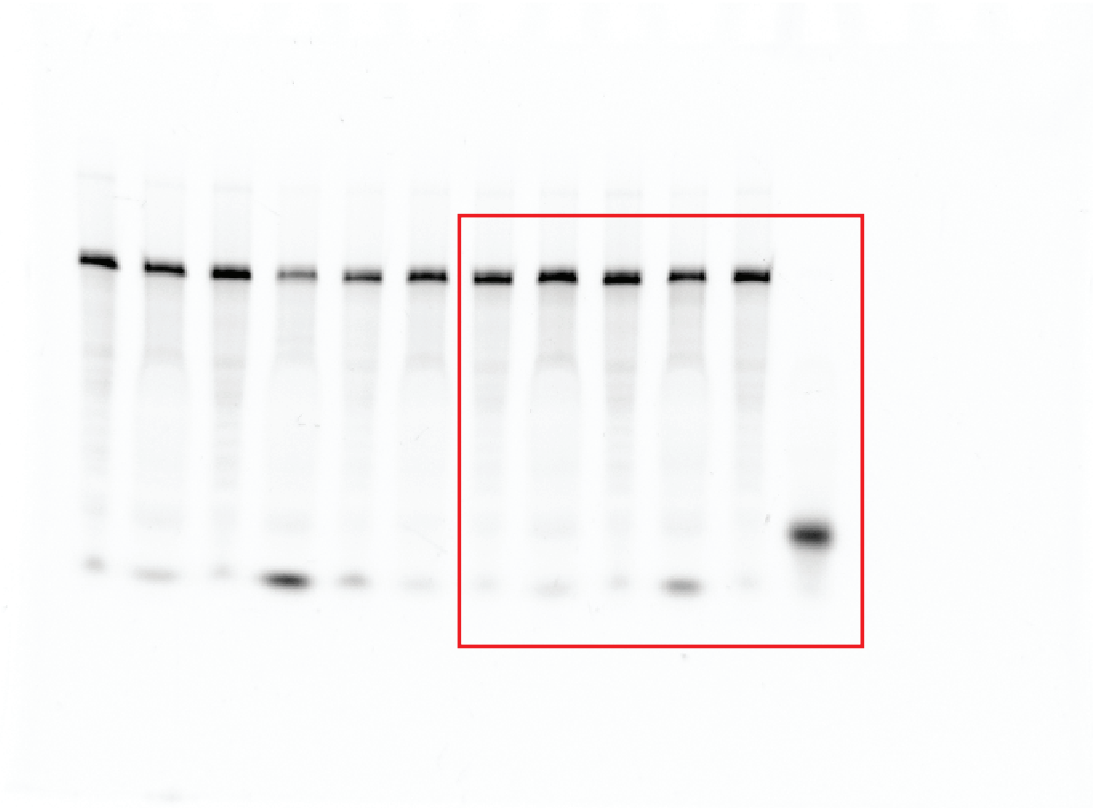

j

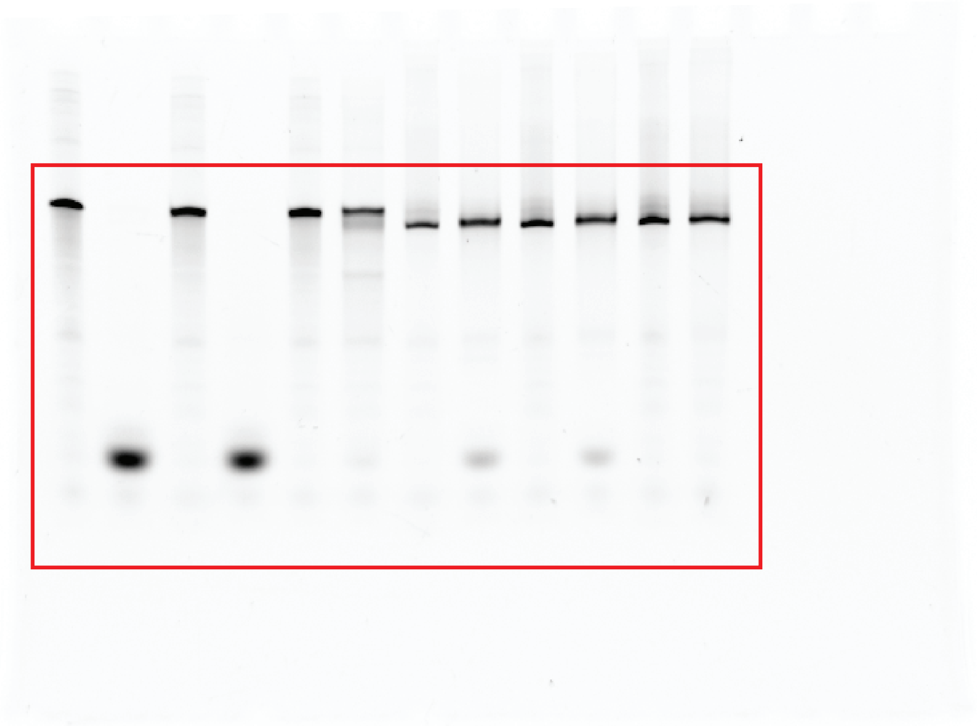

k

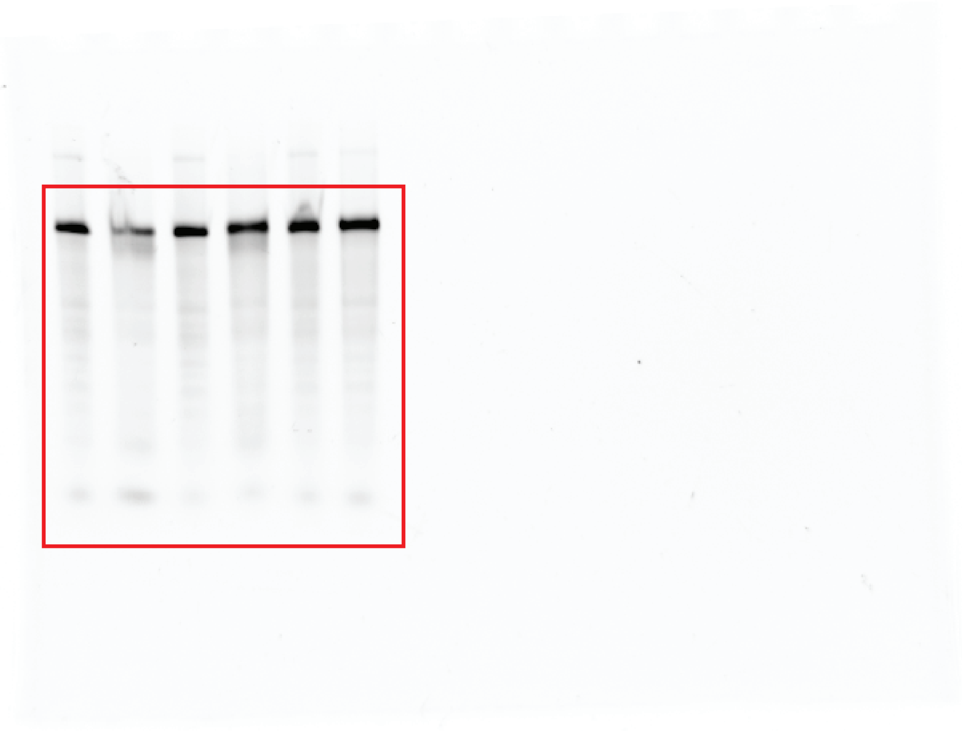

o

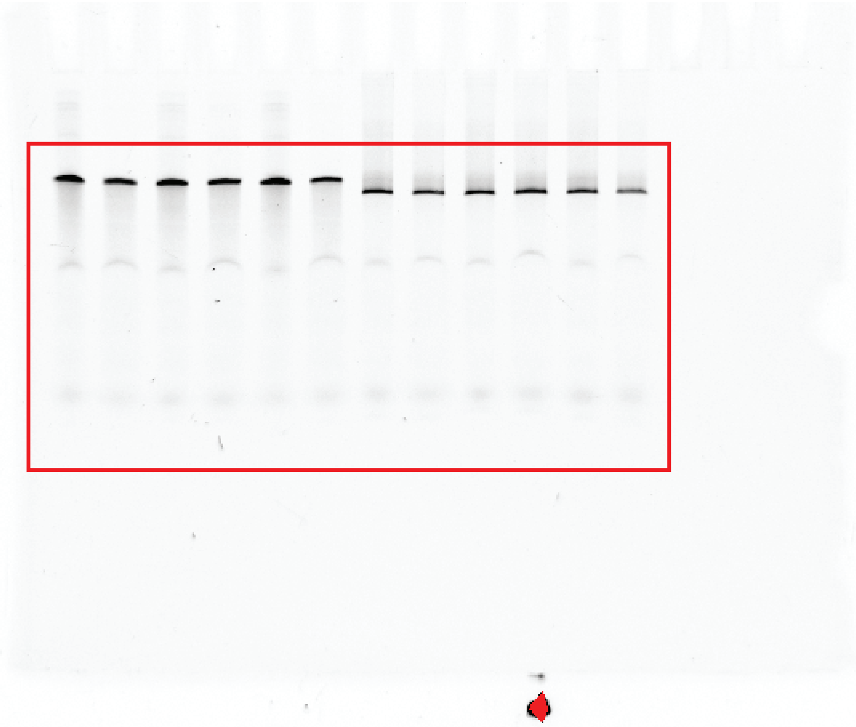

p

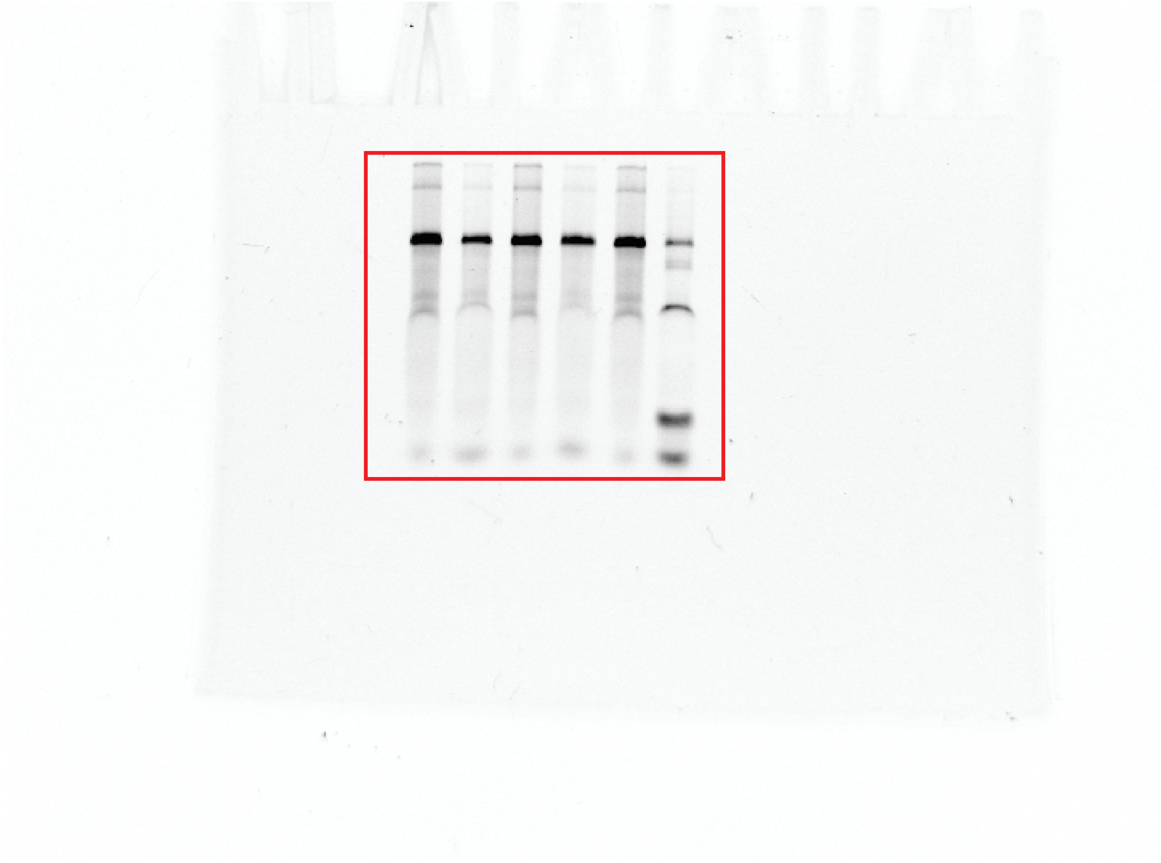

q

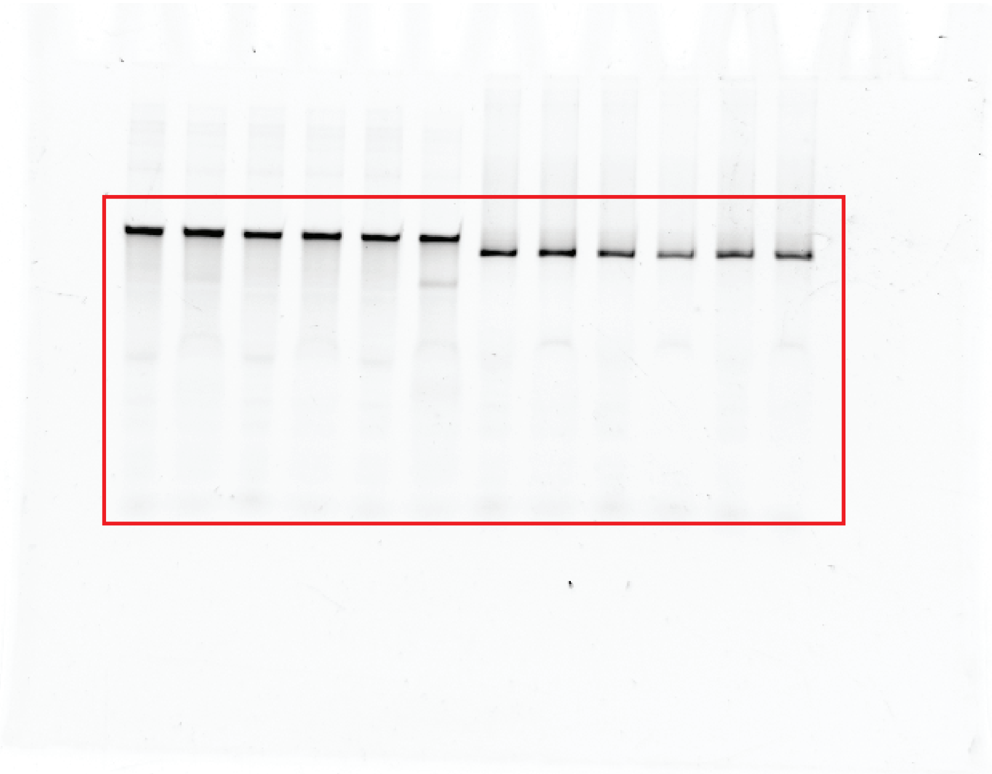

r

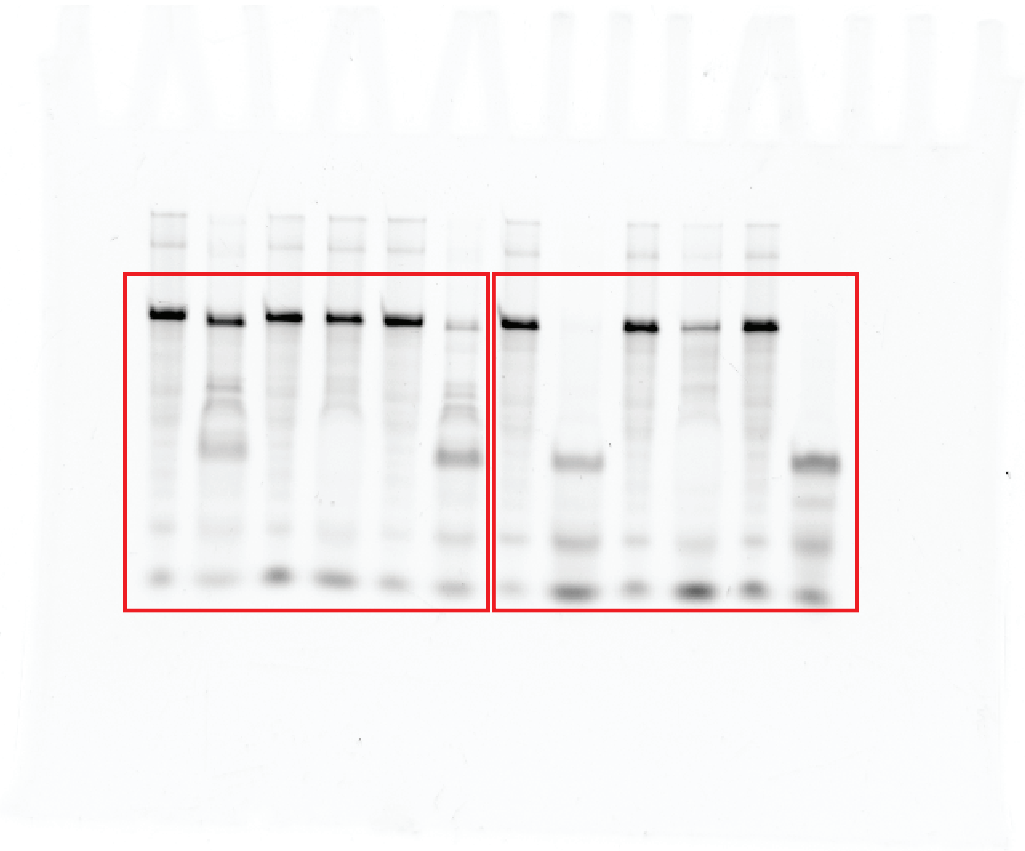

s

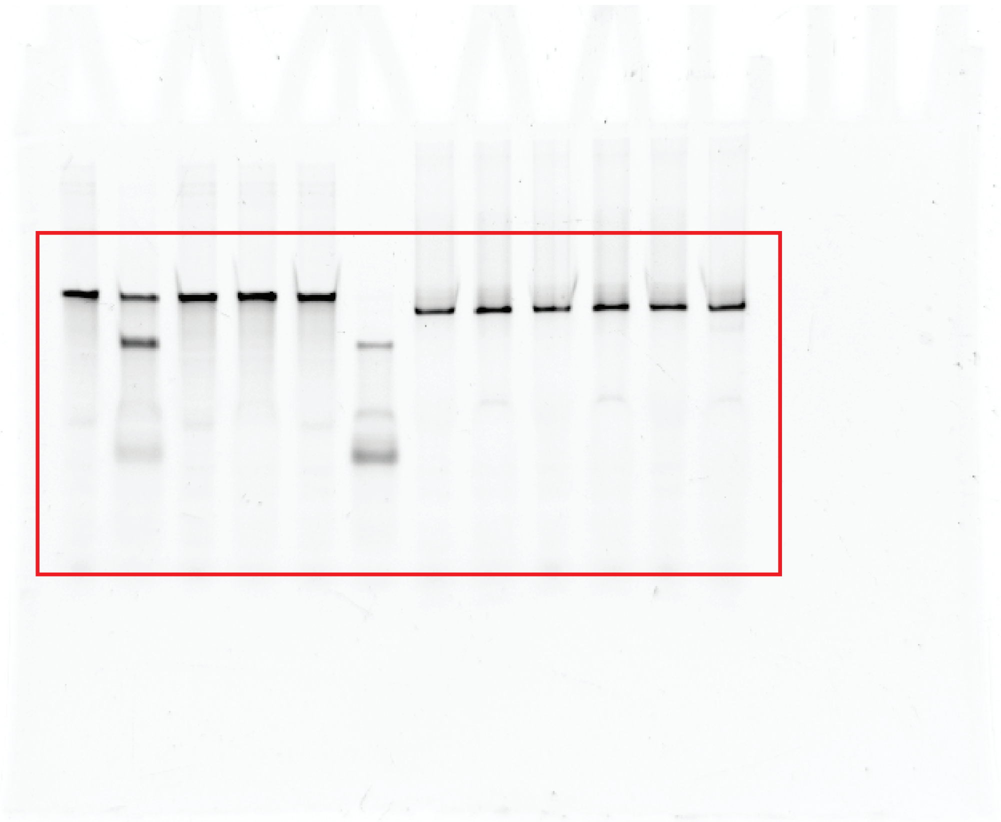

t

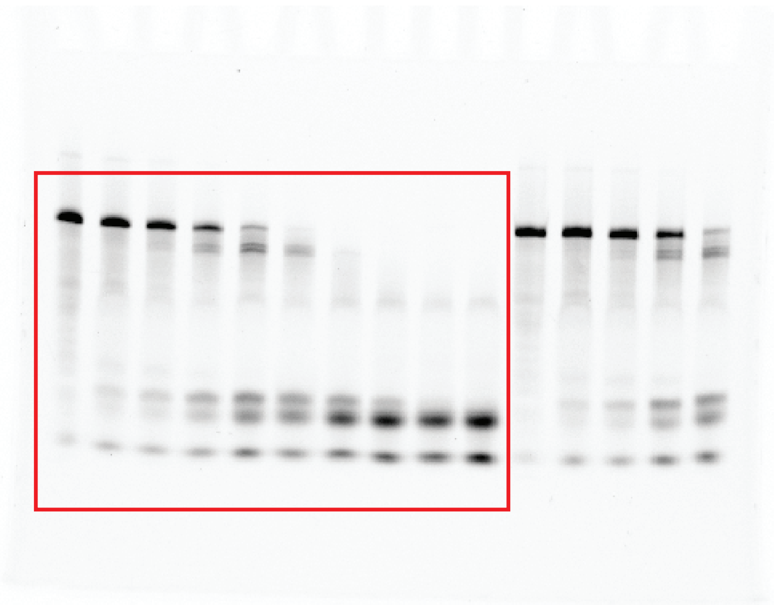

u

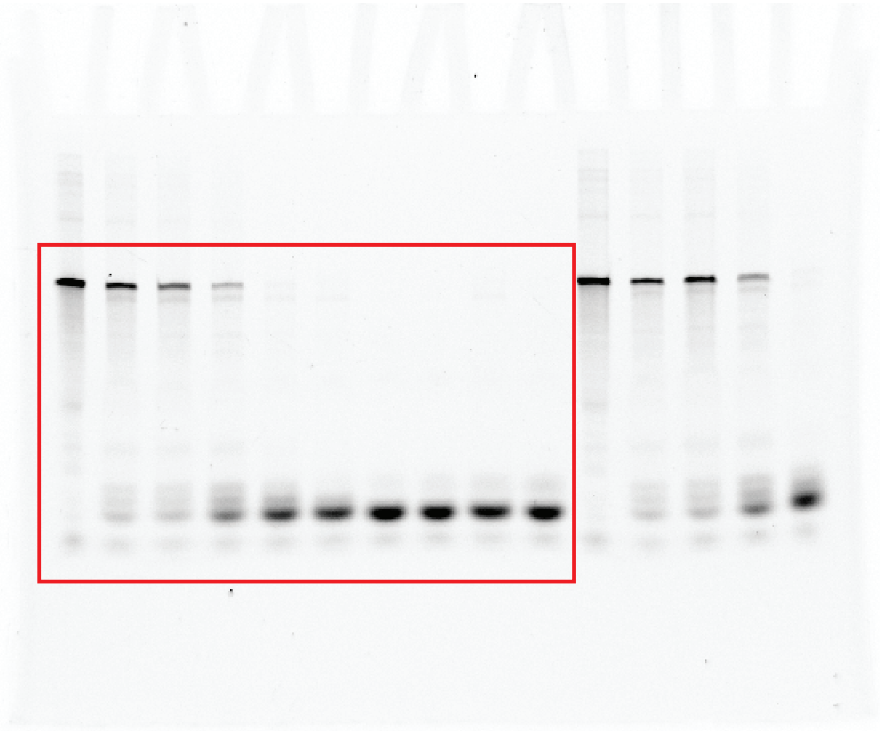

v

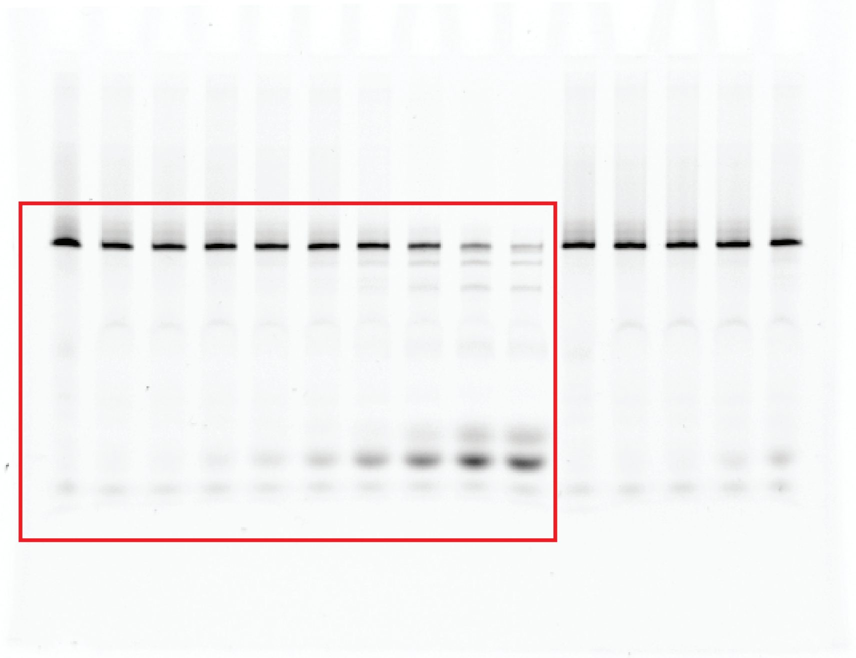

W

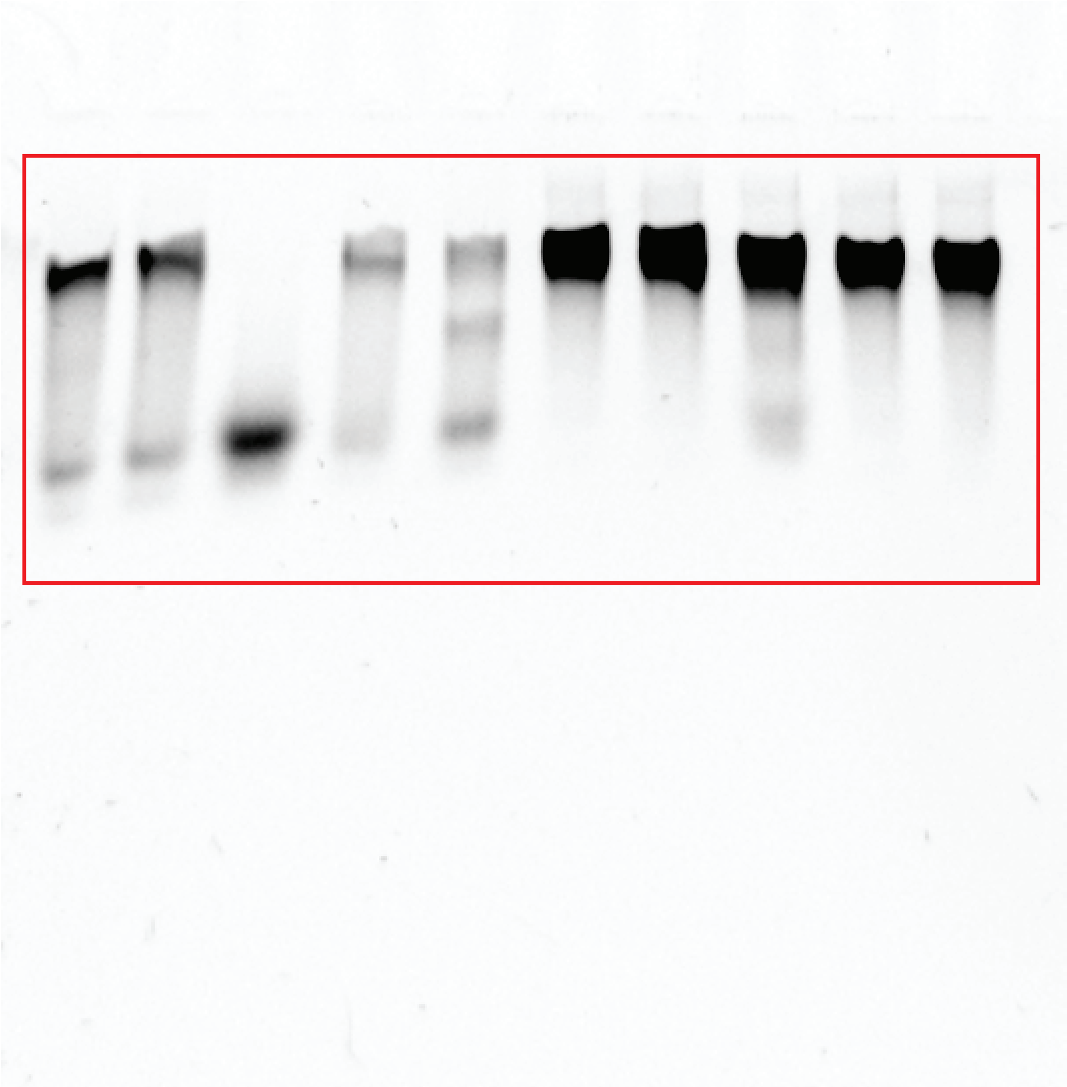

x

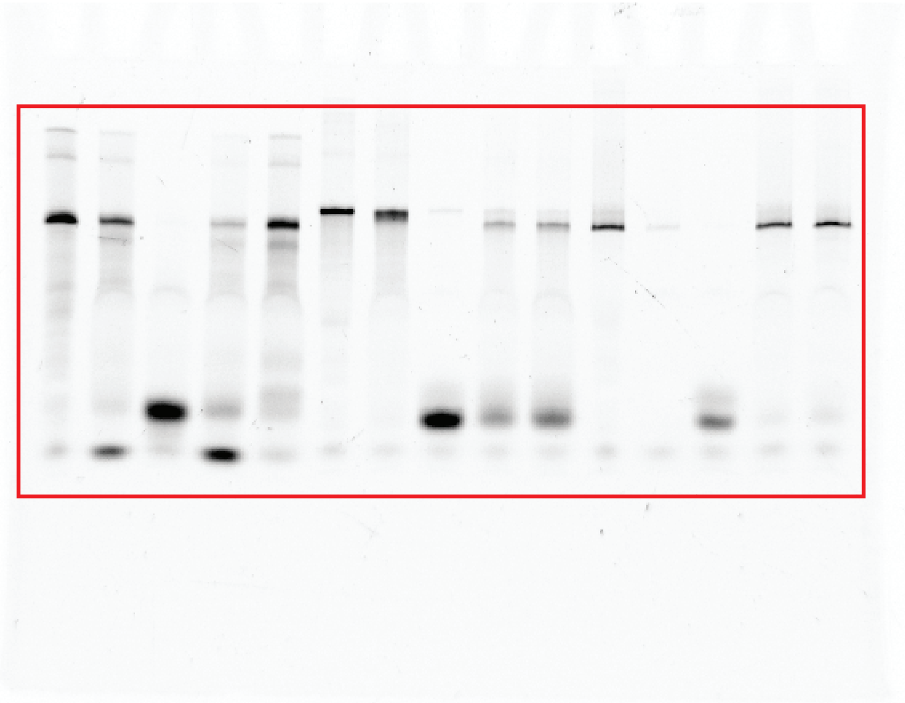

y

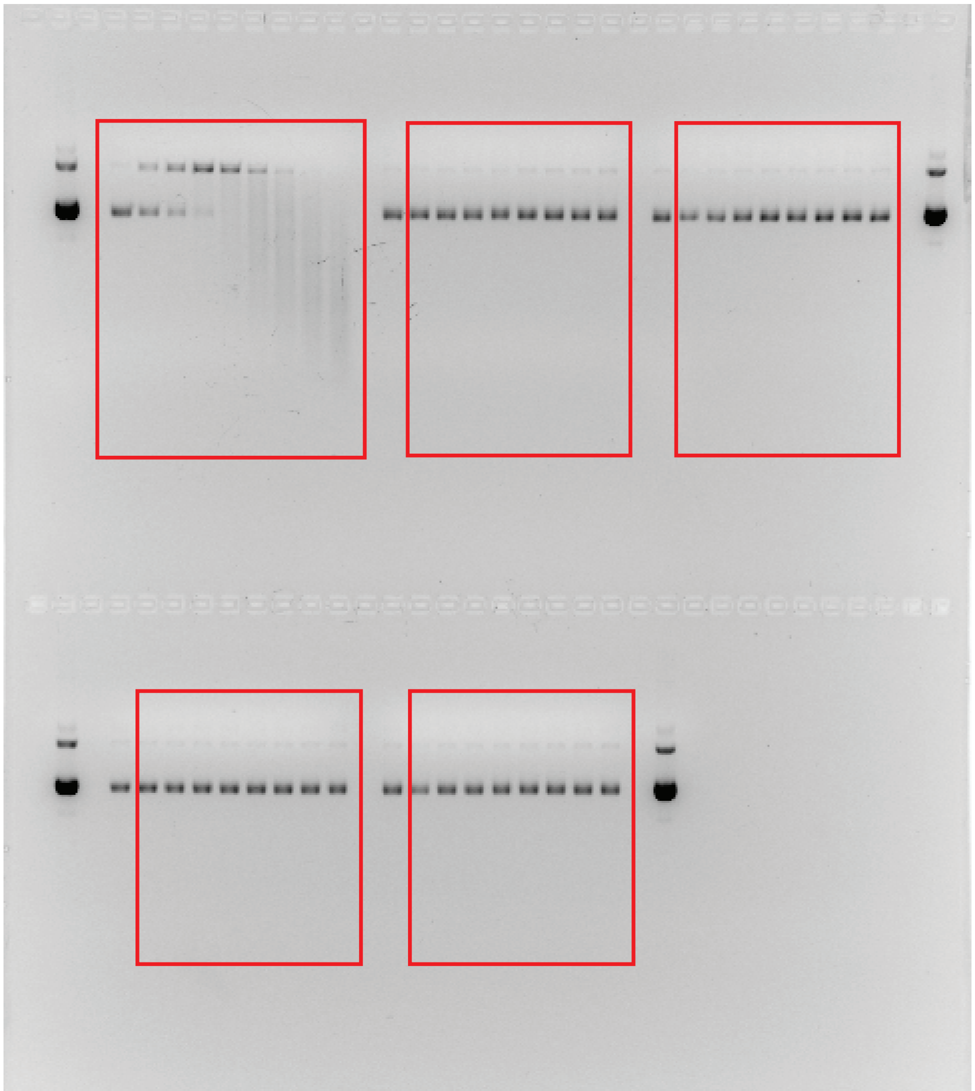

z

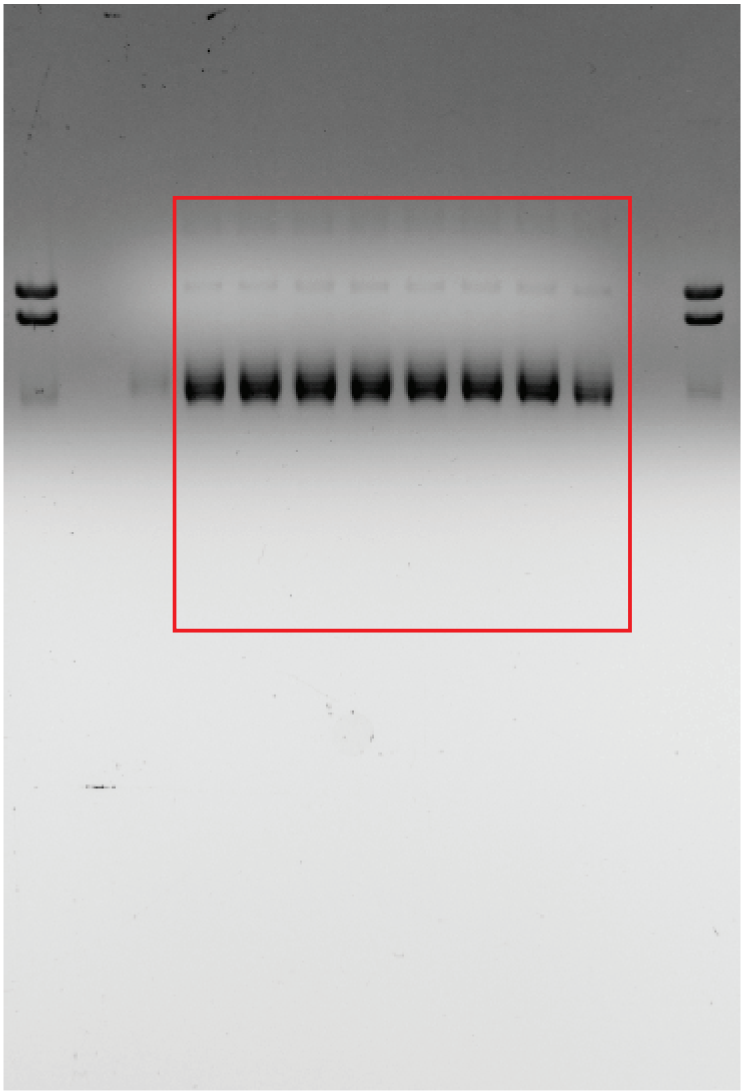

za

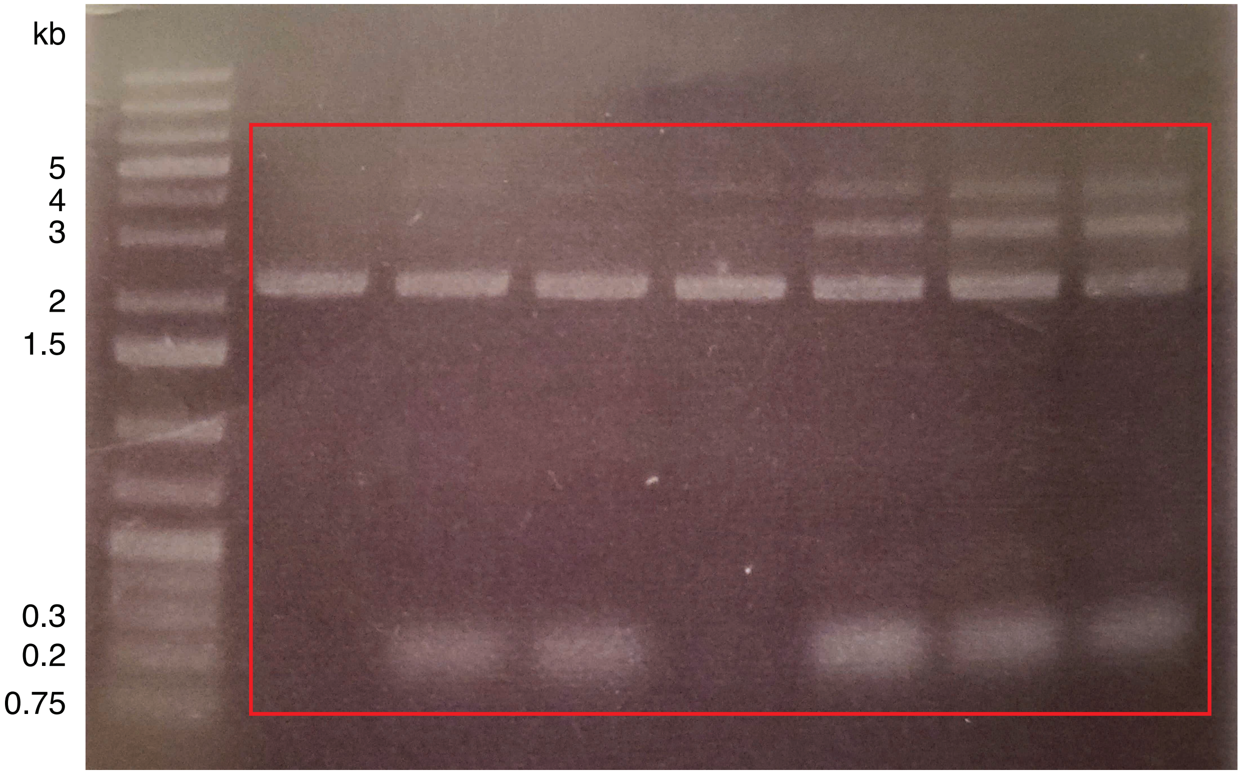

zb

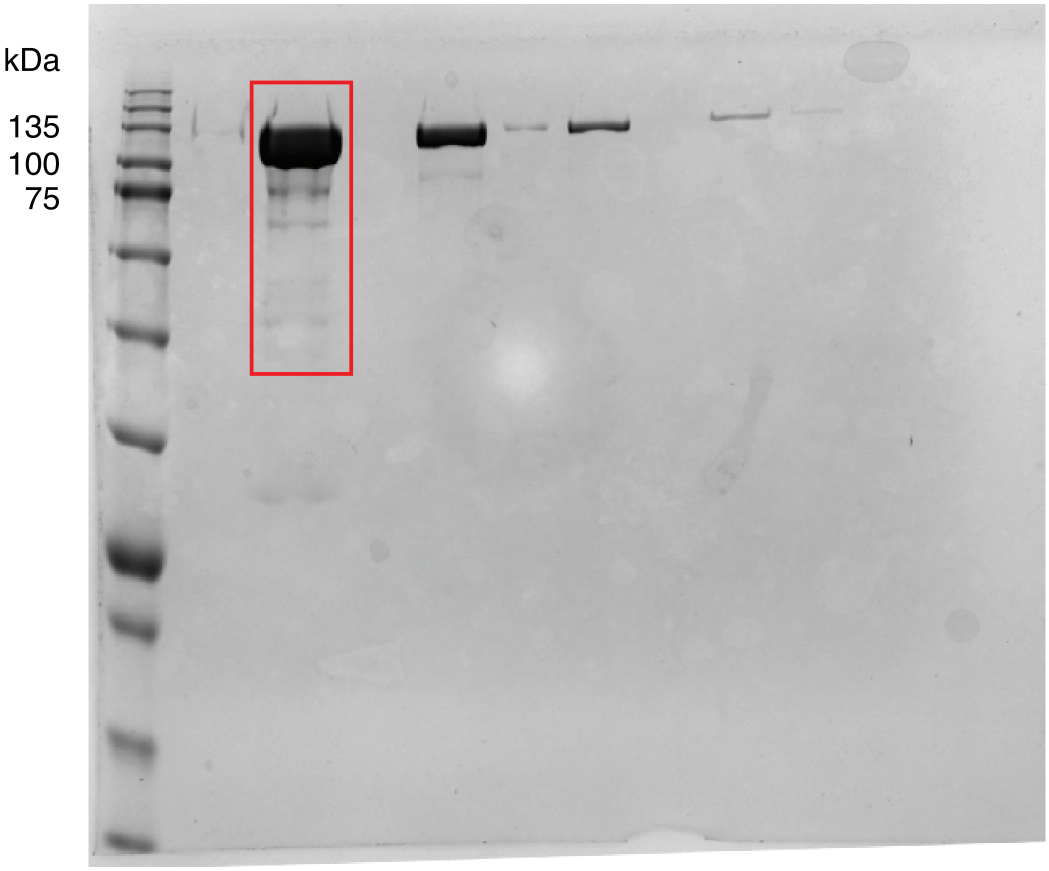

zc

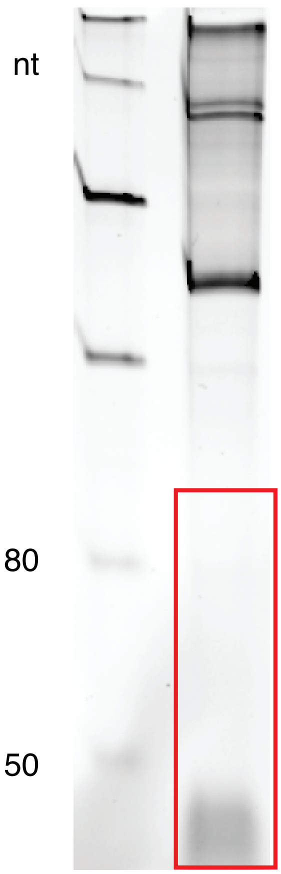

Supplement: Supplementary file 1 — Gel source data. [file 41586_2022_5559_MOESM1_ESM.pdf]
