# Supplementary material for: Cas12a2 elicits abortive infection through RNA-triggered destruction of dsDNA
Source: Nature. 2023 Jan 4;613(7944):588–94. doi: 10.1038/s41586-022-05559-3 (PMC9811890; doi:10.1038/s41586-022-05559-3)
Supplement: Supplementary file 2 — Reporting Summary [file 41586_2022_5559_MOESM2_ESM.pdf]

nature portfolio

Corresponding author(s): Benjamin N. Gray, Ryan N. Jackson, Chase L. Beisel

Last updated by author(s): Nov 4, 2022

Reporting Summary

Nature Portfolio wishes to improve the reproducibility of the work that we publish. This form provides structure for consistency and transparency in reporting. For further information on Nature Portfolio policies, see our Editorial Policies and the Editorial Policy Checklist.

Statistics

For all statistical analyses, confirm that the following items are present in the figure legend, table legend, main text, or Methods section.

n/a	Confirmed	
☐	☒	The exact sample size (*n*) for each experimental group/condition, given as a discrete number and unit of measurement
☐	☒	A statement on whether measurements were taken from distinct samples or whether the same sample was measured repeatedly
☐	☒	The statistical test(s) used AND whether they are one- or two-sided<br>*Only common tests should be described solely by name; describe more complex techniques in the Methods section.*
☒	☐	A description of all covariates tested
☒	☐	A description of any assumptions or corrections, such as tests of normality and adjustment for multiple comparisons
☐	☒	A full description of the statistical parameters including central tendency (e.g. means) or other basic estimates (e.g. regression coefficient) AND variation (e.g. standard deviation) or associated estimates of uncertainty (e.g. confidence intervals)
☐	☒	For null hypothesis testing, the test statistic (e.g. *F*, *t*, *r*) with confidence intervals, effect sizes, degrees of freedom and *P* value noted<br>*Give P values as exact values whenever suitable.*
☒	☐	For Bayesian analysis, information on the choice of priors and Markov chain Monte Carlo settings
☒	☐	For hierarchical and complex designs, identification of the appropriate level for tests and full reporting of outcomes
☒	☐	Estimates of effect sizes (e.g. Cohen's *d*, Pearson's *r*), indicating how they were calculated

Our web collection on statistics for biologists contains articles on many of the points above.

Software and code

Policy information about availability of computer code

Data collection	NovoExpress 1.5.6
Data analysis	Python 3.9, pandas 1.2.0, matplotlib 3.5.2, Excel 16.62, NovoExpress 1.5.6, DBSCAN

For manuscripts utilizing custom algorithms or software that are central to the research but not yet described in published literature, software must be made available to editors and reviewers. We strongly encourage code deposition in a community repository (e.g. GitHub). See the Nature Portfolio guidelines for submitting code & software for further information.

Data

Policy information about availability of data

All manuscripts must include a data availability statement. This statement should provide the following information, where applicable:

- Accession codes, unique identifiers, or web links for publicly available datasets
- A description of any restrictions on data availability
- For clinical datasets or third party data, please ensure that the statement adheres to our policy

The NGS data from the PAM depletion assay and crRNA sequencing data were deposited to NCBI GEO under the accession GSE178536. All other data in the main text or the supplementary materials are available upon reasonable request.

Human research participants

Policy information about studies involving human research participants and Sex and Gender in Research.

Reporting on sex and gender	N/A
Population characteristics	N/A
Recruitment	N/A
Ethics oversight	N/A

Note that full information on the approval of the study protocol must also be provided in the manuscript.

Field-specific reporting

Please select the one below that is the best fit for your research. If you are not sure, read the appropriate sections before making your selection.

☒ Life sciences ☐ Behavioural & social sciences ☐ Ecological, evolutionary & environmental sciences

For a reference copy of the document with all sections, see nature.com/documents/nr-reporting-summary-flat.pdf

Life sciences study design

All studies must disclose on these points even when the disclosure is negative.

Sample size	At least three random colonies were picked as biological replication for each assay. The number of selected colonies were determined based on standards in the field for these types of experiments.
Data exclusions	No data were excluded from the analyses.
Replication	All of the measurements were performed in three, usually four, biological replicates, unless stated otherwise.
Randomization	Random colonies of transformants were picked as biological replicates. Other aspects of randomization (e.g., covariates) are not relevant.
Blinding	Blinding is not relevant to the study, as blinding is not normally included for bacterial and biochemical experiments.

Reporting for specific materials, systems and methods

We require information from authors about some types of materials, experimental systems and methods used in many studies. Here, indicate whether each material, system or method listed is relevant to your study. If you are not sure if a list item applies to your research, read the appropriate section before selecting a response.

Materials & experimental systems

n/a	Involved in the study	
☒	☐	Antibodies
☒	☐	Eukaryotic cell lines
☒	☐	Palaeontology and archaeology
☒	☐	Animals and other organisms
☒	☐	Clinical data
☒	☐	Dual use research of concern

Methods

n/a	Involved in the study	
☒	☐	ChIP-seq
☐	☒	Flow cytometry
☒	☐	MRI-based neuroimaging

Flow Cytometry

Plots

Confirm that:

☒ The axis labels state the marker and fluorochrome used (e.g. CD4-FITC).

☒ The axis scales are clearly visible. Include numbers along axes only for bottom left plot of group (a 'group' is an analysis of identical markers).

☒ All plots are contour plots with outliers or pseudocolor plots.

☒ A numerical value for number of cells or percentage (with statistics) is provided.

Methodology

Sample preparation	For measuring DNA content, the cell pellets were resuspended in 1x PBS containing 2 µg/ml 4',6-diamidino-2-phenylindole (DAPI, ThermoFisher Scientific, 62248). The resuspended cells were stained for 10 minutes in the dark, after which 10 µl were transferred into 240 µl of 1x PBS on a 96 well plate. DAPI fluorescence was measured in the Pacific Blue spectrum (455 nm). Data regarding the forward scatter (FSC) and the side scatter (SSC) were also collected. For live/dead staining, the LIVE/DEAD BacLight Bacterial Viability and Counting Kit (Molecular Probes, L34856) kit was used. The samples were prepared following the kit's manual. Fluorescence was collected in the green (fluorescein for SYTO 9) and red (Texas Red for PI) channels.
Instrument	Cytoflow Novocyte Quanteon flow cytometer
Software	NovoExpress 1.5.6
Cell population abundance	50,000 total ungated events were measured per sample. Pure bacterial cell culture was used for the measurements.
Gating strategy	The dead cells in the live/dead staining experiment in each sample were gated based on the dead-cell suspension control treated with isopropyl alcohol.

☒ Tick this box to confirm that a figure exemplifying the gating strategy is provided in the Supplementary Information.
