# Supplementary material for: Cas12a2 elicits abortive infection through RNA-triggered destruction of dsDNA
Source: Nature. 2023 Jan 4;613(7944):588–94. doi: 10.1038/s41586-022-05559-3 (PMC9811890; doi:10.1038/s41586-022-05559-3)
Supplement: Supplementary file 4 — Supplementary Figs. 2–9 and the legend for Supplementary Fig. 1. [file 41586_2022_5559_MOESM4_ESM.pdf]

SUPPLEMENTARY FIGURES

Supplementary Figure 1 | Gel source data. See the separate PDF file. Raw gel images of Fig. 2a (**a**), Fig. 2b (**b**), Fig. 2d (**c**), Fig. 2e (**d**), Fig. 4c and ED Fig. 7a (**e**), ED Fig. 3c (**f**), ED Fig. 3d (**g**), ED Fig. 4a (**h-s**), ED Fig. 4b (**t-v**), ED Fig. 4c (**w**), ED Fig. 4d (**x**), ED Fig. 4e (**y** and **z**), ED Fig. 10e (**za**), SI Fig. 4b (**zb** and **zc**).

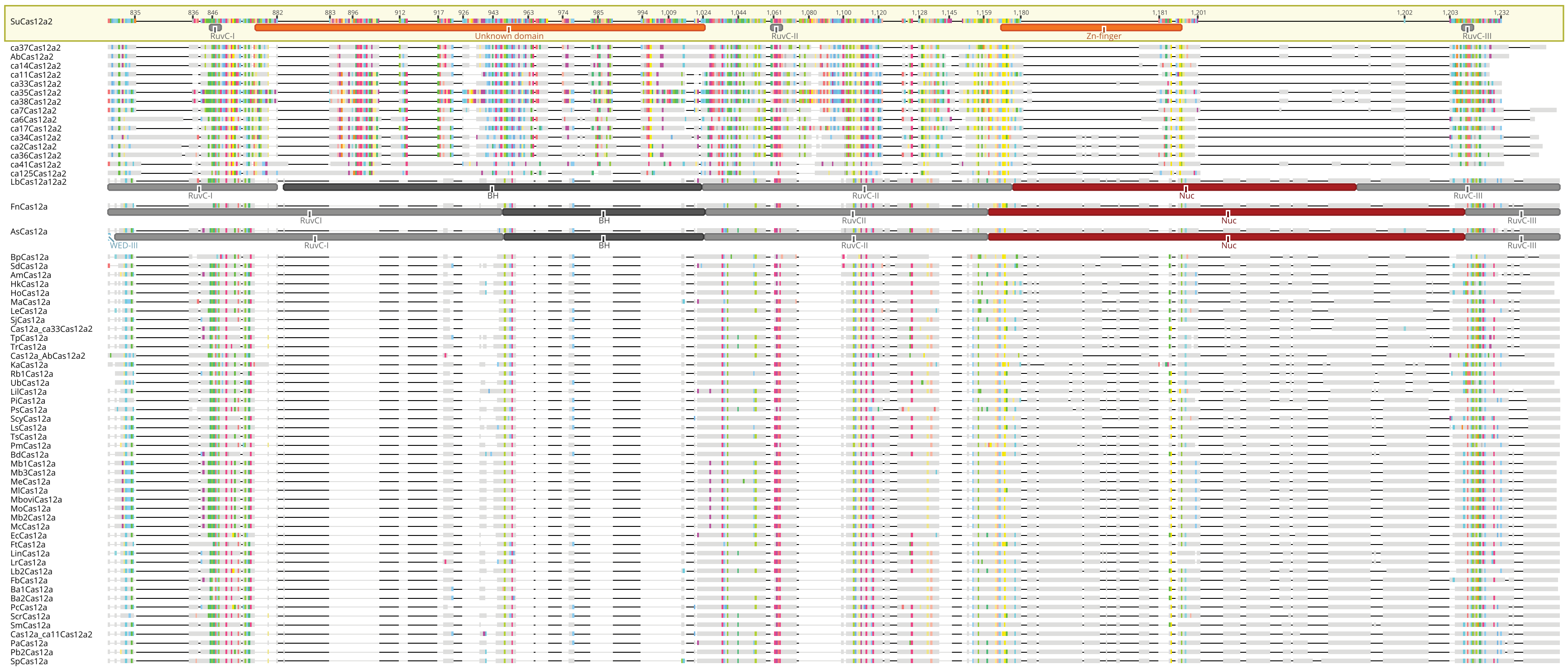

SuCas12a2
835
836
846
882
883
896
912
917
926
943
963
974
985
994
1,009
1,024
1,044
1,061
1,080
1,100
1,120
1,128
1,145
1,159
1,180
1,181
1,201
1,202
1,203
1,232
RuvC-I
Unknown domain
RuvC-II
Zn-finger
RuvC-III
ca37Cas12a2
AbCas12a2
ca14Cas12a2
ca11Cas12a2
ca33Cas12a2
ca35Cas12a2
ca38Cas12a2
ca7Cas12a2
ca6Cas12a2
ca17Cas12a2
ca34Cas12a2
ca2Cas12a2
ca36Cas12a2
ca41Cas12a2
ca125Cas12a2
LbCas12a12a2
RuvC-I
BH
RuvC-II
Nuc
RuvC-III
FnCas12a
RuvCI
BH
RuvCII
Nuc
RuvC-III
AsCas12a
WED-III
RuvC-I
BH
RuvC-II
Nuc
RuvC-III
BpCas12a
SdCas12a
AmCas12a
HkCas12a
HoCas12a
MaCas12a
LeCas12a
SjCas12a
Cas12a_ca33Cas12a2
TpCas12a
TrCas12a
Cas12a_AbCas12a2
KaCas12a
Rb1Cas12a
UbCas12a
LilCas12a
PiCas12a
PsCas12a
ScyCas12a
LsCas12a
TsCas12a
PmCas12a
BdCas12a
Mb1Cas12a
Mb3Cas12a
MeCas12a
MlCas12a
MboviCas12a
MoCas12a
Mb2Cas12a
McCas12a
EcCas12a
FtCas12a
LinCas12a
LrCas12a
Lb2Cas12a
FbCas12a
Ba1Cas12a
Ba2Cas12a
PcCas12a
ScrCas12a
SmCas12a
Cas12a_ca11Cas12a2
PaCas12a
Pb2Cas12a
SpCas12a

Supplementary Figure 2 | Portion of an amino-acid sequence alignment containing the fragmented RuvC domain within Cas12a2 and Cas12a orthologs. The domains in FnCas12a[67], LbCas12a[68], and AsCas12a[69] are based on crystal structures. The domains in SuCas12a2 were predicted using MOTIF search. The highlighted residues represent SuCas12a2 amino acids conserved in other Cas12a2 and Cas12a sequences. Numbering is shown in relation to SuCas12a2.

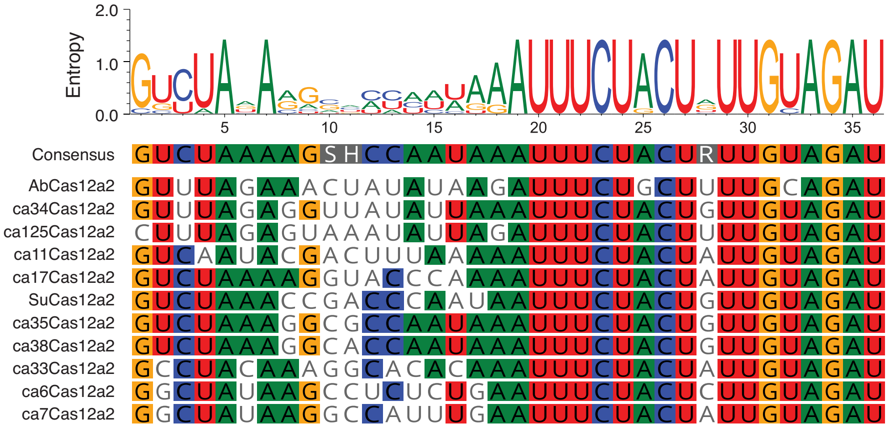

Supplementary Figure 3 | Nucleotide alignment of the direct repeats from CRISPR arrays associated with *cas12a2* genes. Flanking arrays could not be identified for some *cas12a2* genes.

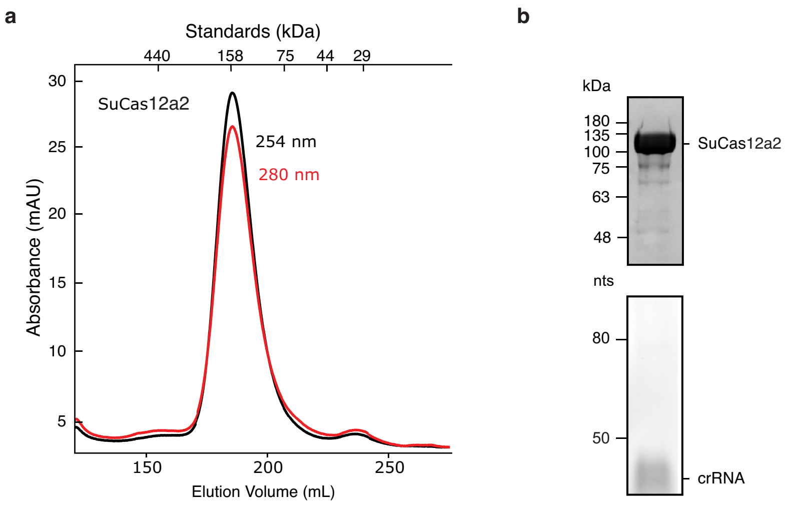

Supplementary Figure 4 | Purification of SuCas12a2. a, Size exclusion chromatogram of purified SuCas12a2 bound to a co-expressed 3X crRNA guide over a Superdex 200 pg 26/600 column. Absorbance was measured at 254 nm and 280 nm. Molecular weight standards are indicated (top). **b**, SDS-PAGE of purified SuCas12a2 + crRNA (top). Urea-PAGE gel of RNA acid-phenol-chloroform extracted from purified SuCas12a2 + crRNA (bottom). For gel source data, see Supplementary Figure 1.

crRNA Processing Loop

```
SuCas12a2WP_041148111.1          -------------KRYGRLQFVCAFNAHIVPQN-17-DVQKRNVTEFNKKVNHAL----SDKEYVVIGIDRGL   851
LnCas12aWP_044910712.1           KVKVLESERVKWSKFYDEQFAVTFSVKKNAD------CLDTTKDLNAEVMEQYSE-------SNRLILIRNT   886
BrCas12aWP_013282991.1           AIKKANEDIIRNRRYTEDKFFLSLSYTKNAD------ISARTLDYINDKVEEDTQD-----SRMAVIVTRNL   892
ParcbacCas12aKKR91555.1          -DGNKEKEVIQHRRFAKDALTLHLKIRLNFG------KHV-NLFDFNKLVNTELFA---KVPVKILGMDRGE   985
EuCAGCas12aWP_022501477.1        GHRKSKIDIVKDKRYTEDKYFLYLPITINYG------IED---ENVNSKIIEYIAK---QDNMNVIGIDRGE   893
EuCas12aWP_012739647.1           EVRTAQKDIVKDYRYTVDKYFIHTPITINYK------VTA--RNNVNDMVVKYIAQ---NDDIHVIGIDRGE   883
MetCas12aWP_015504779.1          KTKKADHDIVKDRRFTVDKMMFHVPIAMNFK------AIS--KPNLNKKVIDGIID---DQDLKIIGIDRGE   851
CanCas12aAIZ56868.1              RYFKAHYDITKDRRYLNDKIYFHVPLTLNFK------ANG--KKNLNKMVIEKFLS---DEKAHIIGIDRGE   862
HcCas12aWP_005398606.1           KYKEARFDIIKDRRYSESQFFFHVPITFNWD------IKT--NKNVNQIVQGMIKD---GEIKHIIGIDRGE   920
AcCas12aWP_021736722.1           ITKEVSHEIIKDRRFTSDKFFFHVPITLNYQ------AAN-SPSKFNQRVNAYLKE---HPETPIIGIDRGE   911
SuccCas12aWP_031492824.1         IKKEATHDITKDKRFTSDKFFFHCPLTINYK------EGD--TKQFNNEVLSFLRG---NPDINIIGIDRGE   937
MiCas12aKKQ38174.1               -----GNKVIDHKRYSENKIFFHVPLTLNRT------KNDSY--RFNAQINNF---LANNKDINIIGVDRGE   894
PcCas12aKKT48220.1               -----GDRAYKYRRYTEKKIMFHMSLVLNTG------KGEIKQVQFNKIINQRISSSDNEMRVNVIGIDRGE   926
AnCas12aWP_027407524.1           KESVFNYDLIKDKRYTERKFYFHCPITLNFR------ADK--PIKYNEKINRFVEN---NPDVCIIGIDRGE   843
ProteoCas12aWP_028830240.1       LKDKFDYPIIKDKRYSQDKFFFHVPMVINYK------SEKLNSKSLNNRTNENLGQ-----FTHIIGIDRGE   784
PdCas12aWP_004356401.1           KVSLFTYDIYKNRRYMENKFLFHLSIVQNYK------AAN-DSAQLNSSATEYIRK---ADDLHIIGIDRGE   946
LnNCCas12aWP_027109509.1         KESIFSYDIVKDKRYTVDKFFIHLPITLNYK------EQN--VSRFNDYIREILKK---SKNIRVIGIDRGE   815
BufbCas12aWP_027216152.1         PTRRLDYDIVKDRRYSQDKFMLHTSIIMNFG------AEE--NVSFNDIVNGVLRN---EDKVNVIGIDRGE   837
OriCas12aWP_049895985.1          KTSTFDYDIVKDRRYCKDKFMLHLPITVNFG------TNE--SGKFNELVNNAIRA---DKDVNVIGIDRGE   834
PseudoCas12aWP_028248456.1       PTSKFGYDIIKDRRYSKDKFMLHIPVTMNFG------VDE--TRRFNDVVNDALRN---DEKVRVIGIDRGE   817
LnmaCas12aWP_044919442.1         ETSTFSYDIVKDKRYSKDKFTLHIPITMNFG------VDE--VKRFNDAVNSAIRI---DENVNVIGIDRGE   818
FnCas12a_3WP_004339290.1         KESVFEYDLIKDKRFTEDKFFFHCPITINFK------SSG--ANKFNDEINLLLKE--KANDVHILSIDRGE   927
PmCas12aWP_018359861.1           ETSLFNYDLVKDKRFTEDKFFFHVPISINYK------NKK--ITNVNQMVRDYIAQ---NDDLQIIGIDRGE   863
MxCas12aKDN25524.1               -KRQFVYDIIKDKRYTQDKFMLHVPITMNFG------VQGMTIKEFNKKVNQSIQQ---YDEVNVIGIDRGE   989
LpCas12aWP_020988726.1           LFEKLKYPILKDKRYSEDKFQFHLPISLNFK------SKE--RLNFNLKVNEFLKR---NKDINIIGIDRGE   876
SmCas12aWP_037385181.1           ATSTFNYDIVKDKRYTIDKFQFHVPITMNFK------AEG--IFNMNQRVNQFLKA---NPDINIIGIDRGE   866
FbCas12aCCB70584.1               NEKNKTIDIIKDKRFTVDKFQFHVPITMNFK------ATG--GSYINQTVLEYLQN---NPEVKIIGLDRGE   932
FlavbCas12aWP_045971446.1        AKNTFAYDLIKDKRYTVDKFQFHVPITMNFK------ATG--NSYINQDVLAYLKD---NPEVNIIGLDRGE   888
LnMCCas12aWP_044910713.1         EVSVFPYDIIKNRRYTVDKFQFHVPLKMNFK------ADE--KKRINDDVIEAIRS---NKGIHVIGIDRGE   842
LnNDCas12aWP_035635841.1         KTTTLSYDVYKDKRFSEDQYELHIPIAINKC------PKN--IFKINTEVRVLLKH---DDNPYVIGIDRGE   835
LnCoeCas12aWP_016301126.1        KESMFDYDIIKDKRFTCDKYQFHVPITMNFK------ALG--ENHFNRKVNRLIHD---AENMHIIGIDRGE   845
PorpCas12aWP_023936172.1         EESLFEYDLVKDRRYTMDKFQFHVPITMNFK------CSA--GSKVNDMVNAHIRE---AKDMHVIGIDRGE   883
PrevbCas12aWP_044110123.1        KQSNFEYDLIKDKRYTVDKFMFHVPITLNFK------GMG--NGDINMQVREYIKT---TDDLHFIGIDRGE   877
BoCas12aWP_009217842.1           KESKFEYDLIKDRRYTVDKFLFHVPITMNFK------SVG--GSNINQLVKRHIRS---ATDLHIIGIDRGE   868
PvCas12aWP_024988992.1           HTSTFKYDIIKDRRYTVDKFQFHVPITINFK------ATG--QNNINPIVQEVIRQ---NGITHIIGIDRGE   865
PbB_Cas12aEFI70750.1             KESIFDYDLVKDKRYTVDKFQFHVPITMNFK------STG--NTNINQQVIDYLRT---EDDTHIIGIDRGE   869
                                              **
```

Positively Charged Active Site Residues

Supplementary Figure 5 | Clustal Omega amino acid sequence alignment of SuCas12a2 with Cas12a sequences. Cas12a amino acids located within the loop involved in crRNA-processing are indicated in red. Conserved positively charged residues involved in processing[20] are indicated. Although the putative crRNA-processing loop of Cas12a2 is not as long, the positively charged residues appear to be conserved.

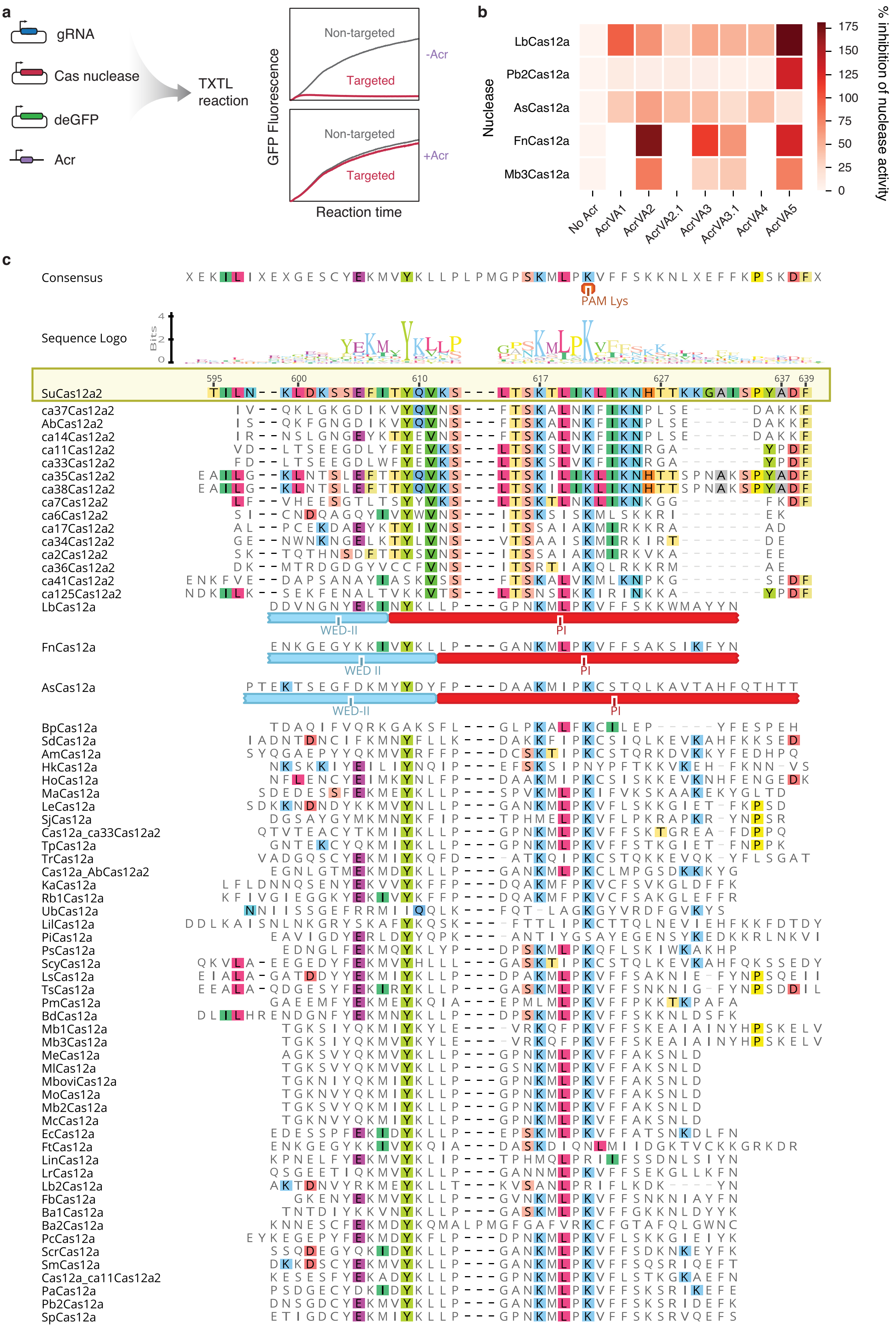
a
gRNA
Cas nuclease
deGFP
Acr
TXTL reaction
GFP Fluorescence
Non-targeted
Targeted
-Acr
+Acr
Reaction time
b
Nuclease
LbCas12a
Pb2Cas12a
AsCas12a
FnCas12a
Mb3Cas12a
No Acr
AcrVA1
AcrVA2
AcrVA2.1
AcrVA3
AcrVA3.1
AcrVA4
AcrVA5
% inhibition of nuclease activity
175
150
125
100
75
50
25
0
c
Consensus
PAM Lys
Sequence Logo
Bits
SuCas12a2
WED-II
PI
WED II
LbCas12a
FnCas12a
AsCas12a

Supplementary Figure 6 | Verification of DNA targeting inhibition by AcrVA proteins in TXTL. **a**, Schematic of the Acr targeting inhibition assay in TXTL. **b**, Percent inhibition of Cas12a activity by the known type V-A anti-CRISPR proteins in TXTL. Inhibition above 100% reflects higher GFP levels for the target versus the non-target reaction in the presence of a given Acr. The mean of at least three biological replicates are shown. **c**, Amino-acid sequence alignment of the locus containing the lysine residue acetylated by AcrVA5 in Cas12a[39]. The alignment shows that this residue is also present in Cas12a2 orthologs. The highlighted residues represent conserved amino acids relative to SuCas12a2. Numbering is shown in relation to SuCas12a2. The domains in FnCas12a[67], LbCas12a[68], and AsCas12a[69] are based on crystal structures.

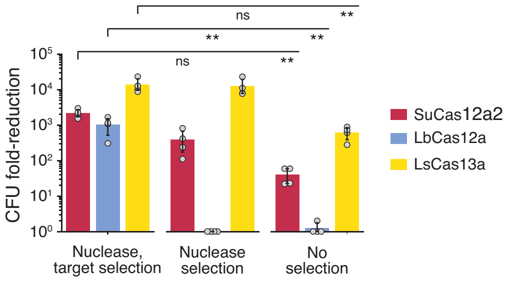

Supplementary Figure 7 | Reduction in colony-forming units (CFU) following nuclease and crRNA induction under different antibiotic selection conditions in *E. coli* BL21(AI). See Fig. 4a for the corresponding time course data. Values are means ± s.t.d. of 4 independent experiments started from separate colonies. ns: $p > 0.05$, *: $p < 0.05$, **: $p < 0.005$ calculated with one-tailed Welch's t-test.

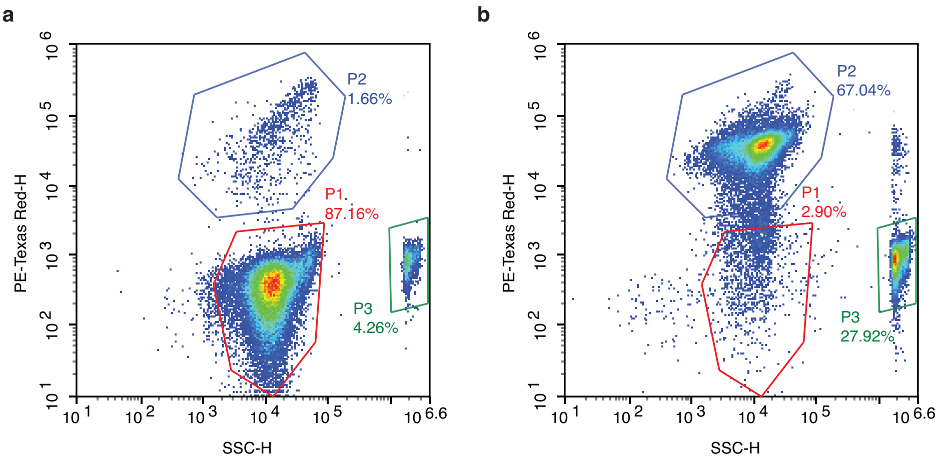

Supplementary Figure 8 | Examples of the gating strategy used in Figure 4b. a, A sample used to gate live cells (P1). **b**, A sample used to gate dead cells treated with isopropanol (P2). Gate P3 encompasses beads supplied with the LIVE/DEAD BacLight™ Bacterial Viability and Counting Kit (Molecular Probes, L34856).

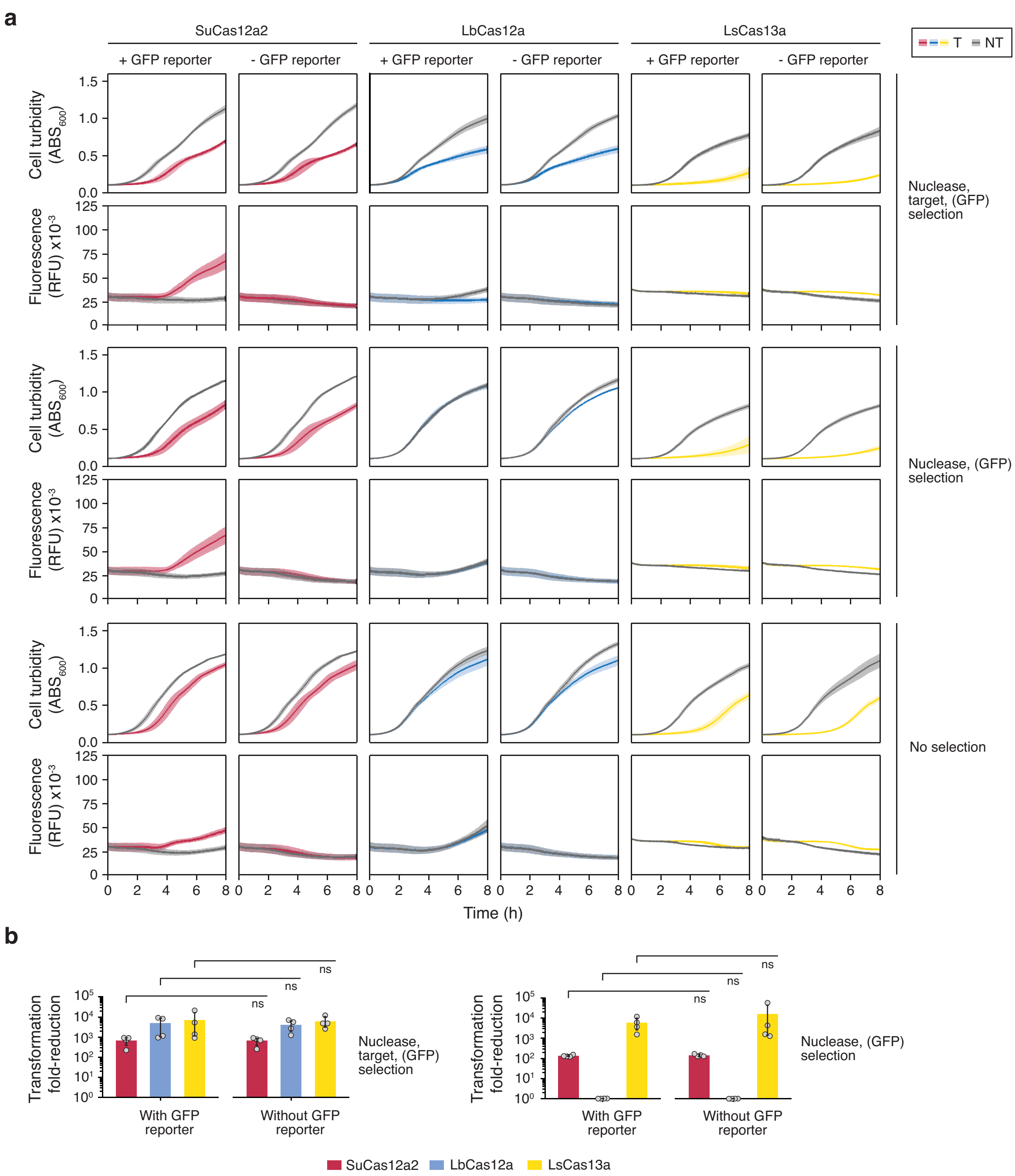

Supplementary Figure 9 | Inclusion of the SOS reporter construct does not perturb plasmid interference by the different Cas nucleases. a, Turbidity and fluorescence time course measurements when assessing the SOS-responsive GFP reporter under different antibiotic selection conditions in *E. coli* BL21(AI). Darker bands represent the mean values of four independent experiments. The shaded areas depict one standard deviation from the mean. **b**, Impact of the SOS-responsive GFP reporter on plasmid interference in *E. coli* BL21(AI) under different selection conditions. Bar heights represent mean values of four independent experiments. Values are means ± s.t.d. of 4 independent experiments started from separate colonies. ns: $p > 0.05$, *: $p < 0.05$, **: $p < 0.005$ calculated with one-tailed Welch's t-test.
